# Albumin prevents kidney injury but is underutilized in a cohort of patients undergoing large-volume paracentesis

**DOI:** 10.1097/HC9.0000000000000760

**Published:** 2025-09-29

**Authors:** Rahul Rajkumar, Nikki Duong, W. Ray Kim, Elisabet Viayna, Thomas Ardiles, Cristina Coll-Ortega, E. Anne Davis, Jonathan Lilley, Xuan Zhang, Nisha Wadhwani, Kunal Lodaya

**Affiliations:** 1 Boston Strategic Partners, Inc., Boston, Massachusetts, USA; 2 Division of Gastroenterology and Hepatology, Stanford University School of Medicine, Redwood City, California, USA; 3 Division of Gastroenterology and Hepatology, Mayo Clinic College of Medicine and Science, Phoenix, Arizona, USA; 4 Global Health Economics and Outcomes Research & Real World Evidence (GHEOR & RWE), Grifols, Sant Cugat del Vallès, Barcelona, Spain; 5 Grifols, SSNA, Research Triangle Park, Durham, North Carolina, USA; 6 Cencora, Conshohocken, Pennsylvania, USA

**Keywords:** decompensated cirrhosis, pharmacoequity, real-world evidence, social determinants of health, structural racism

## Abstract

**Background:**

Cirrhosis and cirrhosis-related deaths have risen in the United States in recent years. Ascites is a common complication, often requiring large-volume paracentesis (LVP). The American Association for the Study of Liver Diseases (AASLD) recommends the administration of albumin in conjunction with LVP to prevent further complications of cirrhosis. Emerging research in cirrhosis care reveals significant variations in outcomes among different demographics. Therefore, we assessed the use of guideline-adherent albumin and outcomes in U.S. patients undergoing LVPs, particularly at the intersection of race, ethnicity, socioeconomic disparities, and cirrhosis.

**Methods:**

This retrospective study utilized Cerner Real World Data to identify adults with cirrhosis and ascites undergoing LVP between January 2016 and June 2022. We assessed albumin utilization patterns across racial and ethnic groups and payor types, and their overall impact on acute kidney injury (AKI)-related hospitalization using an adjusted generalized linear model (aGLM).

**Results:**

We identified 736 patients: 301 in the LVP + albumin group and 435 in the LVP-only group. Despite clinical recommendations, only 41% undergoing LVPs received albumin. White patients and commercially insured patients received albumin at higher rates (*p*=0.042 and *p*=0.009, respectively). The overall rate of AKI-related admissions within the 30-day post-procedure period was 26%. However, patients who received albumin during LVP had a 36% lower risk of short-term AKI-related hospitalization (OR: 0.64; *p*=0.03).

**Conclusions:**

These findings indicate a potential for broader albumin utilization in U.S. patients with refractory ascites undergoing repeated LVPs to reduce AKI-related admissions.

## INTRODUCTION

Cirrhosis is a leading cause of morbidity and mortality in the United States, where it accounts for 150,000+ hospitalizations annually and over $4 billion in healthcare costs attributed to decompensating events, re-admissions, or non-adherence to cirrhosis-related quality indicators.^1^^–^^3^ Ascites is the most common complication of decompensated cirrhosis and is typically managed with a combination of dietary sodium restriction and diuretics as first-line management.^4^ In up to 10% of patients, ascites becomes refractory to medical therapy due to the inability to mobilize fluid despite maximal doses of diuretics or due to complications such as acute kidney injury (AKI) and hyponatremia.^5^ Refractory ascites (RA) is important to recognize since it has been associated with survival rates as low as 50% at 6 months.^6^ Patients with RA may require TIPS, liver transplantation (LT), or most commonly, repeated large-volume paracentesis (LVP).^7^^–^^9^

While LVPs provide symptomatic relief in patients with RA, post-paracentesis circulatory dysfunction (PPCD) is a potential complication due to the reduction of effective arterial blood volume.^10^ Given that maintaining effective blood volume is crucial for kidney function, patients undergoing frequent LVPs are more susceptible to AKI.^11^^,^^12^ AKI in the setting of decompensated cirrhosis portends an ominous prognosis. In-hospital mortality in patients with cirrhosis has been reported to be 6-fold higher among those with AKI as compared with those without AKI.^13^ Hence, patients with RA undergoing paracentesis require careful monitoring to identify AKI onset, followed by early intervention.^14^^,^^15^

The American Association for the Study of Liver Diseases (AASLD) recommends the infusion of human albumin in conjunction with LVPs to prevent PPCD and related complications including renal impairment, owing to albumin’s intravascular volume expansion properties to preserve effective arterial blood volume and its hypothesized role in controlling systemic inflammation.^5^^,^^16^^–^^20^

Recent studies have also suggested variations in cirrhosis outcomes to be potentially linked to race and ethnicity, highlighting the importance of understanding the interplay of health disparities, social determinants of health, and cirrhosis.^1^^,^^2^^,^^21^^–^^23^ However, the evidence linking race and ethnicity to cirrhosis outcomes is conflicting, necessitating further research. One study found higher re-admissions in non-White patients with cirrhosis than White patients, while another reported no racial impact on 30-day mortality.^21^^,^^23^ Studies are required to evaluate albumin utilization in the context of RA and its clinical outcomes in the United States, particularly at the intersection of race and ethnicity, socioeconomic disparities, and cirrhosis.

Therefore, we utilized real-world data from a large U.S. electronic health record (EHR) database to investigate albumin utilization among patients with cirrhosis and ascites undergoing LVPs. We aimed to report albumin usage trends across different races and ethnicities and payor types, as well as their impact on mortality and the incidence of AKI.

## METHODS

### Data source

In this retrospective observational study, we utilized Cerner Real-World Data, a de-identified, HIPAA-compliant database of EHR from over 700 participating clinical facilities and hospital systems across the United States.

### Ethics approval and governance

Ethics approval and informed consent were waived by the Western Institutional Review Board (Puyallup, WA) based on a predefined statistical analysis plan (Ref. #1-1418180-1; approved March 31, 2021), in accordance with 45 CFR § 46.104(d). The research utilized anonymized data, documents, and pre-existing records only, with no prospective data collection. All research was conducted in accordance with both the Declarations of Helsinki and Istanbul.

### Study population

We identified adults ≥18 years old with a diagnosis of cirrhosis based on current procedural terminology codes and the International Classification of Diseases, Tenth Revision (ICD-10) codes (Supplemental Table S1, http://links.lww.com/HC9/C104) between January 2016 and June 2022. Using these codes, we queried the database for patients with a diagnosis of ascites who underwent at least 2 LVPs within 12 months during the study period, and those who underwent 2 LVPs within 30 days, ensuring the inclusion of patients with RA. Additional criteria for inclusion required LVPs to occur within 24 hours of hospital intake. Patients with a hospital length of stay ≥48 hours or a concomitant diagnosis of hepatorenal syndrome or spontaneous bacterial peritonitis were excluded.

### Exposure, outcomes, and covariates

We categorized patients into 2 groups: those who received albumin in conjunction with LVP (“LVP + albumin”), and those who underwent LVP without albumin (“LVP only”). We evaluated patient demographics and self-reported race/ethnicity (White or Caucasian, Hispanic, Native American or Pacific Islander, Black or African American, Asian, Other, or Mixed). We focused on multiple payor groups, including Medicaid, Medicare, Commercial, Self-pay, TRICARE, Government, and Charity. Hospital characteristics, patient characteristics at index, and cirrhosis etiology were also evaluated.

We assessed albumin utilization patterns across racial and ethnic groups and various payor categories and their overall impact on mortality and AKI-related and HE-related inpatient admissions within 30 days of index LVP using corresponding ICD-10 codes indicating admitting diagnoses (Supplemental Table S1, http://links.lww.com/HC9/C104). LT within 12 months of index LVP and healthcare resource utilization metrics within 30 days from LVP were also evaluated.

### Statistical analysis

Continuous variables were expressed as means (SD) and medians (IQR), while categorical variables were expressed as frequencies and proportions of the overall cohort and across study groups. *p-*values were reported for numeric and categorical data using Kruskal–Wallis and chi-square tests, respectively, and values <0.05 were considered significant. We conducted a logistic regression analysis to assess the association between various predictors and the receipt of albumin. The impact of albumin receipt on AKI-related hospitalization was assessed using an adjusted generalized linear model (aGLM). Data were analyzed using a combination of Jupyter Notebook (version 7), SQL (version 16.0.4015.1), Python (version 3.11), and Pandas (version 2.0).

## RESULTS

### Study participants

**FIGURE 1:**
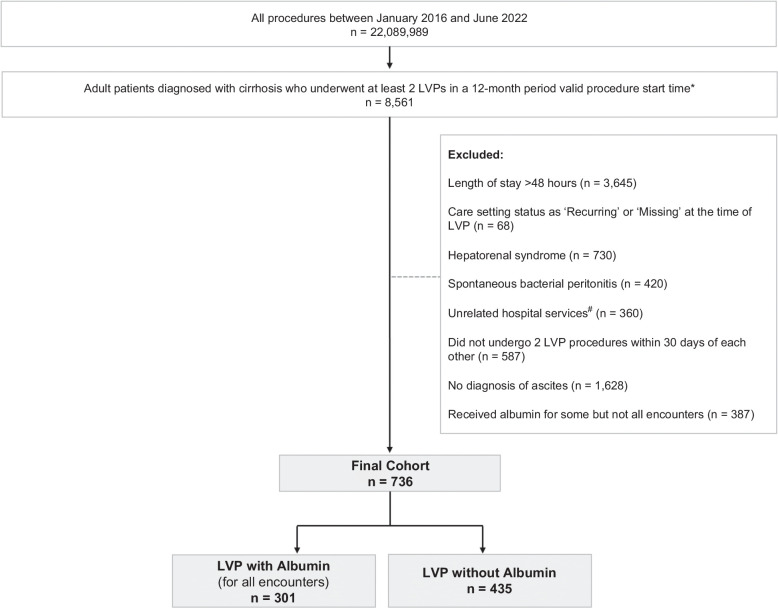
Patient flowchart. *Within 24 hours of admission and ≤ discharge time. ^#^Exclusionary services, for example, cardiology, obstetrics, and neurology. Abbreviations: LVP, Large-volume paracentesis.

Between January 2016 and June 2022, we identified records from 52,232 patients who underwent an LVP. Of these, 8561 had a cirrhosis diagnosis and at least 2 LVPs within 12 months. Applying further exclusion criteria yielded our final cohort of 736 patients with ascites secondary to cirrhosis who underwent 2 LVPs within 30 days, of whom 301 received albumin and 435 did not receive albumin (Figure 1).

### Overall patient and hospital characteristics at first LVP (index)

The overall mean age was 56.7 (11.4) years, with 67.9% being male, and 81.4% being treated at teaching hospitals. The majority of patients were identified as White or Caucasian (68.1%), while the most common payor type was Medicaid (36.1%). Alcohol-associated liver disease (ALD) was the most common etiology of cirrhosis (74.5%), and the mean Charlson Comorbidity Index (CCI) was 3.8 (2.7) (Table 1).

**TABLE 1:** Characteristics of patients during the first encounter

	Overall (n=736)	LVP + Albumin (n=301)	LVP only (n=435)	*p*
Patient characteristics				
Age (y)				
Median (IQR)	57.0 (50.0, 64.0)	58.0 (52.0, 65.0)	56.0 (49.0, 63.0)	0.002
Mean±SD	56.7±11.4	58.2±11.0	55.6±11.5	
Sex, n (%)				
Male	500 (67.9)	214 (71.1)	286 (65.7)	0.147
Female	235 (31.9)	86 (28.6)	149 (34.3)	0.122
Unspecified	1 (0.1)	1 (0.3)	0 (0.0)	0.853
Race, n (%)				
White or Caucasian	501 (68.1)	218 (72.4)	283 (65.1)	0.042
Hispanic	155 (21.1)	63 (20.9)	92 (21.1)	1.000
Native American or Pacific Islander	34 (4.6)	10 (3.3)	24 (5.5)	0.224
Black or African American	28 (3.8)	8 (2.7)	20 (4.6)	0.247
Asian	7 (1.0)	1 (0.3)	6 (1.4)	0.292
Mixed	5 (0.7)	1 (0.3)	4 (0.9)	0.619
Other	6 (0.8)	0 (0)	6 (1.4)	0.103
Payor group, n (%)				
Medicaid	266 (36.1)	99 (32.9)	167 (38.4)	0.147
Medicare	169 (23.0)	73 (24.3)	96 (22.1)	0.546
Commercial	137 (18.6)	70 (23.3)	67 (15.4)	0.009
Self	26 (3.5)	14 (4.7)	12 (2.8)	0.244
Other^a^	21 (2.9)	10 (3.3)	11 (0.2)	0.681
Unspecified	117 (15.9)	35 (11.6)	82 (18.9)	0.011
Hospital characteristics				
Region,^b^ n (%)				
Midwest	33 (4.5)	7 (2.3)	26 (6.0)	0.030
Northeast	61 (8.3)	31 (10.3)	30 (6.9)	0.131
South	96 (13.0)	37 (12.3)	59 (13.6)	0.695
West	546 (74.2)	226 (75.1)	320 (73.6)	0.706
Bed size, n (%)				
<100	5 (0.7)	2 (0.7)	3 (0.7)	1.000
100–199	1 (0.1)	0 (0.0)	1 (0.2)	1.000
200–299	54 (7.3)	24 (8.0)	30 (6.9)	0.684
300–499	76 (10.3)	19 (6.3)	57 (13.1)	0.004
500–999	64 (8.7)	36 (12.0)	28 (6.4)	0.013
1000+	536 (72.8)	220 (73.1)	316 (72.6)	0.961
Teaching hospital, n (%)	599 (81.4)	247 (82.1)	352 (80.9)	0.768
Care setting at index encounter, n (%)				
Emergency	114 (15.5)	33 (11.0)	81 (18.6)	0.007
Inpatient	179 (24.3)	69 (22.9)	110 (25.3)	0.517
Observation	14 (1.9)	5 (1.7)	9 (2.1)	0.901
Outpatient	397 (53.9)	194 (64.5)	203 (46.7)	<0.001
Unspecified	32 (4.4)	0 (0)	32 (7.3)	<0.001
Clinical characteristics				
Charlson comorbidity index				
Median (IQR)	4.0 (1.0, 5.0)	4.0 (1.0, 5.0)	4.0 (1.0, 5.0)	0.369
Mean±SD	3.8±2.7	3.9±2.7	3.7±2.6	
Etiology of cirrhosis,^c^ n (%)				
Alcohol-associated liver disease	548 (74.5)	222 (73.8)	326 (74.9)	0.781
Hepatitis C	75 (10.2)	24 (8.0)	51 (11.7)	0.126
Hepatitis B	17 (2.3)	7 (2.3)	10 (2.3)	1.000
MASLD/MASH	152 (20.7)	58 (19.3)	94 (21.6)	0.498

^a^ Includes TRICARE, Government, and Charity payor types.

^b^ The first digit of the zip code was used (0, 1=Northeast, 2, 3, 7=South, 4–6=Midwest, 8, 9=West).

^c^ Non-mutually exclusive.

Abbreviations: CCI, Charlson comorbidity index; MASH, metabolic dysfunction–associated steatohepatitis; MASLD, metabolic dysfunction–associated steatotic liver disease.

### Patient characteristics across both LVP groups (index)

**FIGURE 2:**
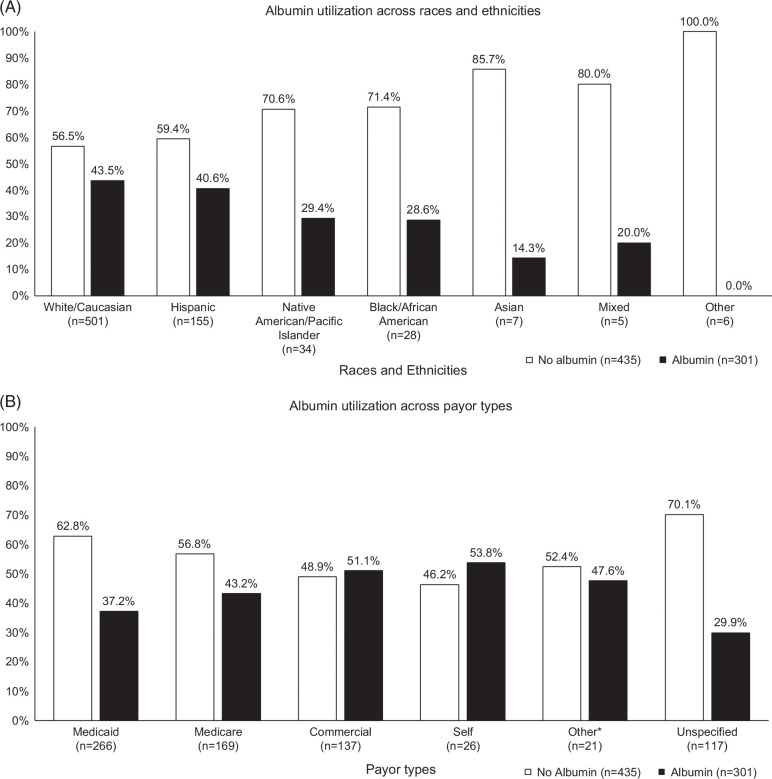
Albumin utilization across (A) races and ethnicities and (B) payor types. *Includes TRICARE, Government, and Charity payor types.

Only 41% (n=301) consistently received albumin at the time of LVP. A higher proportion of White and commercially insured patients received albumin (*p*=0.042 and *p*=0.009, respectively). In comparison to other races and insurance groups, the descriptive trends suggest that White and Hispanic patients (Figure 2A), and those with commercial and self-insurance (Figure 2B) received albumin more frequently. More than half of all LVPs (64.5%) performed in the outpatient setting involved the conjunctive use of albumin, which was surprisingly less frequent in inpatient (22.9%), observation (1.7%), and emergency (11%) settings.

### Associations with albumin receipt using logistic regression

The logistic regression analyses indicate that patients who presented in emergency departments (ED) experienced a 52% decrease in the likelihood of receiving albumin (OR: 0.48, 95% CI: 0.30–0.77, *p*=0.003) compared with outpatients, with no significant associations observed among Black or Hispanic patients (Table 2). The “other” group including patients with various racial and ethnic minorities, had a 56% reduced likelihood of receiving albumin compared with White patients (OR: 0.44, 95% CI: 0.22–0.90, *p*=0.024).

**TABLE 2:** Logistic regression model predicting albumin receipt

Predictor	Odds ratio (95% CI)	*p*
Age group		
≤50	Reference	
51–57	1.15 (0.73–1.81)	0.549
58–64	1.27 (0.80–2.03)	0.306
≥65	2.10 (1.17–3.77)	0.013
Sex		
Male	Reference	
Female	0.77 (0.54–1.10)	0.155
Race and ethnicity		
White	Reference	
Black	0.69 (0.27–1.72)	0.424
Hispanic	0.99 (0.65–1.51)	0.957
Other^a^	0.44 (0.22–0.90)	0.024
Teaching hospital status		
Yes	0.99 (0.60–1.62)	0.954
Care setting		
Outpatient	Reference	
Emergency	0.48 (0.30–0.77)	0.003
Inpatient	0.72 (0.48–1.07)	0.100
Observation	0.49 (0.15–1.61)	0.240
Region		
West	Reference	
Midwest	0.64 (0.23–1.79)	0.395
Northeast	1.61 (0.89–2.94)	0.118
South	1.17 (0.66–2.08)	0.588
CCI group		
0–2	Reference	
3–4	0.82 (0.55–1.22)	0.317
≥5	1.30 (0.85–1.97)	0.224
Payor group		
Medicaid	Reference	
Medicare	0.68 (0.40–1.18)	0.174
Commercial	1.53 (0.96–2.42)	0.072
Self	1.85 (0.79–4.36)	0.159
Other^b^	1.07 (0.41–2.80)	0.893
Unspecified	0.53 (0.30–0.92)	0.025
Alcohol-associated liver disease		
Yes	0.97 (0.66–1.42)	0.866
Hepatitis C		
Yes	0.66 (0.38–1.14)	0.133
Hepatitis B		
Yes	1.22 (0.43–3.52)	0.708
MASLD/MASH		
Yes	0.83 (0.56–1.24)	0.366

^a^ Includes Native American or Pacific Islander, Asian, Mixed, and Other races.

^b^ Includes TRICARE, Government, and Charity payor types.

Abbreviations: CCI, Charlson comorbidity index; MASH, metabolic dysfunction–associated steatohepatitis; MASLD, metabolic dysfunction–associated steatotic liver disease.

### Follow-up characteristics and patient-level outcomes within 30 days of the first LVP

Twenty-four percent of patients in the LVP + albumin group had an AKI-related admission compared with 28% in the LVP-only group (*p*=0.205) (Table 3). The 30-day mortality rate was 2.0% in the LVP + albumin group compared with 2.8% in the LVP-only group (*p*=0.675). The rate of LT within 12 months of index LVP was 5.3% and 3.9% in the LVP + albumin versus the LVP-only group (*p*=0.467), respectively.

**TABLE 3:** Follow-up characteristics within 30 days of the index large-volume paracentesis

	Overall(n=736)	LVP + albumin (n=301)	LVP only(n=435)	*p*
Inpatient admissions/incidence of complications				
AKI-related admission, n (%)	193 (26.2)	71 (23.6)	122 (28.0)	0.205
HE-related admission, n (%)	2 (0.3)	1 (0.3)	1 (0.2)	1.000
Mortality within 30 d from the last LVP encounter, n (%)	18 (2.4)	6 (2.0)	12 (2.8)	0.675
Liver transplant within 12 mo of index, n (%)	33 (4.5)	16 (5.3)	17 (3.9)	0.467
Patient-level outcomes				
Total hospital days, n (%)	470 (63.9)	192 (63.8)	278 (63.9)	
Median (IQR)	3.4 (1.3, 7.6)	3.5 (1.1, 7.5)	3.3 (1.4, 7.6)	0.805
Mean±SD	5.7±6.2	5.7±6.6	5.6±6.0	
Inpatient days, n (%)	379 (51.5)	144 (47.8)	235 (54.0)	
Median (IQR)	5.0 (2.6, 8.9)	4.8 (2.8, 8.8)	5.0 (2.6, 9.0)	0.415
Mean±SD	6.5±5.4	6.8±6.0	6.3±5.0	
Inpatient visits, n (%)	379 (51.5)	144 (47.8)	235 (54.0)	
Median (IQR)	1.0 (1.0, 2.0)	1.0 (1.0, 1.0)	1.0 (1.0, 2.0)	0.160
Mean±SD	1.3±0.6	1.3±0.6	1.4±0.6	
Outpatient visits, n (%)	561 (76.2)	254 (84.4)	307 (70.6)	
Median (IQR)	3.0 (2.0, 5.0)	3.0 (2.0, 6.0)	3.0 (2.0, 5.0)	0.101
Mean±SD	3.9±3.0	4.1±2.8	3.7±3.1	
Emergency room visits, n (%)	268 (36.4)	88 (29.2)	180 (41.4)	
Median (IQR)	1.0 (1.0, 2.0)	1.0 (1.0, 2.0)	1.0 (1.0, 2.0)	0.143
Mean±SD	1.7±1.1	1.6±0.9	1.8±1.2	

Abbreviations: AKI, acute kidney injury; LVP, large-volume paracentesis.

### Association of albumin administration with AKI-related hospitalization within 30 days following first LVP

**FIGURE 3:**
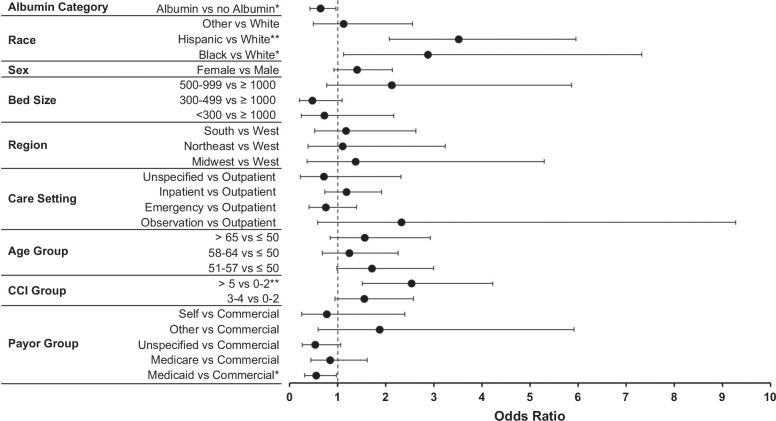
Adjusted generalized linear model for AKI-related inpatient admissions within 30 days of index LVP. **p<0.01 and *p<0.05. Adjusted for age, sex, race and ethnicity, payor type, U.S. region, hospital bed size, care setting and CCI. For race: other includes Native American or Pacific Islander, Asian, Mixed, and Other races. For Payor group: other includes TRICARE, Government, and Charity payor types. Abbreviations: AKI, acute kidney injury; CCI, Charlson comorbidity index; LVP, large-volume paracentesis.

Given the direct link between PPCD and AKI in the absence of albumin, we delve into the impact of albumin infusion on the occurrence of AKI by regression analysis. In our aGLM, patients in the LVP + albumin group had a 36% decrease in the odds of AKI-related hospitalizations (OR: 0.64, 95% CI: 0.42–0.96, *p*=0.030) (Figure 3).

Moreover, compared with their White counterparts, Black and Hispanic individuals were 2.9 and 3.5 times more likely, respectively, to have an AKI-related hospitalization (OR: 2.87, 95% CI: 1.12–7.32, *p*=0.030 and OR: 3.51, 95% CI: 2.07–5.95, *p*<0.001, respectively). Patients with a CCI score of 5+ had 2.5 times higher odds of an AKI-related hospitalization (OR: 2.53, 95% CI: 1.51–4.22, *p*<0.001) compared with those with a score of 0–2. Medicaid-insured patients were less likely to have AKI-related admission (OR: 0.55, 95% CI: 0.31–0.97, *p*=0.040) compared with commercial payors.

## DISCUSSION

In this retrospective observational cohort study of patients with cirrhosis and ascites who underwent 2 or more LVPs within 30 days, only 41% received albumin at the time of LVP, despite clinical recommendations. ALD was the most prevalent cause of cirrhosis in the majority of patients. A higher proportion of White and commercially insured patients received albumin. One-fourth of all patients with RA required an AKI-related admission within 30 days of LVP. This is unsurprising, as LVP is a common cause of AKI in decompensated cirrhosis due to an accelerated pathological hemodynamic change secondary to an intravascular contraction and a decrease in renal perfusion.^24^^,^^25^ However, patients who received albumin along with an LVP conferred a 36% reduction in risk of an AKI-related hospitalization.

In this study, ALD emerged as the most frequent cause of cirrhosis, followed by MASLD/MASH. These have emerged as primary drivers of end-stage liver disease over the last decade.^1^ In the United States, alcohol misuse has become the primary cause of chronic liver disease,^26^ which worsened with the COVID-19 pandemic, with significant long-term impacts.

Differences in albumin use across racial and ethnic groups may reflect underlying structural inequities that contribute to variations in care delivery. This aligns with prior studies reporting inequities in transplant referral patterns and waitlist outcomes among racially and ethnically minoritized populations, including Hispanic, Asian, American Indian/Alaskan Native, and Black ALD patients.^27^ These inequities extend into post-transplant outcomes as well; for example, UNOS data from 2016 also show that Black patients experience higher 5-year post-transplant mortality compared with White patients.^28^ However, our analysis did not stratify racial and ethnic groups by insurance payor type or disease severity, which may influence albumin administration. Future research is warranted to explore the impact of these and other clinically relevant factors that were beyond the scope of our analysis.

We also observed varying rates of albumin receipt descriptively among different payor types in our study. Despite Medicaid comprising the largest proportion of the cohort’s payor type (36%), only 37% received albumin along with their LVPs, whereas over half of all commercially and self-insured patients received albumin. This is an interesting finding, especially considering that widespread initiatives were carried out to expand healthcare access, increase advancements in treatment, and enhance efforts to improve care quality through the extension of Medicaid.^29^^–^^32^ Our data also indicates that only 43% of Medicare-insured patients received albumin, compared with 51% of those with commercial insurance and 54% of self-insured patients. While we cannot exclude the role of systemic or institutional factors, these observed differences may also be due to clinical considerations not captured in our dataset, such as higher rates of comorbidities, including cardiac disease, or the risk of volume overload. Further research is needed to better understand the clinical and nonclinical factors influencing albumin use in this population.

Similar disparities were observed with Medicaid recipients facing higher denial rates for chronic HCV treatment and a 4-fold increase in 90-day hospital re-admissions.^33^ The authors point out that at baseline, Black patients were more likely to have Medicaid, which independently correlated with re-admission. A 2009–2018 HCUP-NIS database study reported that White patients were least likely to have Medicaid, while Black and Hispanic patients were least likely to receive TIPS or LT.^31^ In a study of Medicare enrollees with HE, Black patients were the least likely to receive early Rifaximin prescription and had limited access to gastroenterology care than White patients, with Hispanic patients facing similar barriers in access to consultation.^34^

Our regression analysis also revealed a 52% lower likelihood of receiving albumin in ED compared with outpatient settings, likely attributed to the ED’s emphasis on immediate stabilization, unlike outpatient settings that prioritize long-term care. Consequently, treatment disparities may contribute to higher re-admissions and pharmaco-inequities, potentially driven by costs or implicit biases at both the provider and system level. These findings indicate that the differences in albumin use across care settings may reflect more than just patient characteristics or insurance type, including potential differences in provider specialty, familiarity with guideline-based care, and workflow constraints across settings. While our study was not designed to directly assess provider-level or system-level drivers, this remains an important area for future research.

Although albumin offers several potential benefits including ligand binding, anti-inflammatory, antioxidant, and endothelial stabilization effects which remain to be validated in RCTs, its prohibitive cost may have led to its underutilization in this study.^17^^,^^18^^,^^21^ While it is recommended to infuse 6–8 g of albumin per liter of ascites removed above 5 L (pending pivotal dose–response studies),^5^^,^^17^^,^^18^ one could argue that lower doses of albumin could be considered in patients undergoing LVP to address the potential cost issue. Hussain et al's^35^ evaluation of low-dose albumin (4 g/L removed) confirmed its safety and efficacy in preventing PPCD with similar rates of renal dysfunction. Underutilization may also be attributed to a lack of provider training on the updated guidelines. Changes to the EHR could mitigate this issue, as shown by Anderson et al,^36^ where the integrated order set resulted in a reduction of 1.8 g/L of albumin administered, remained safe, and saved $30,000 annually. This concept, however, warrants prospective evaluation to explore its potential to standardize practices and reduce racial and ethnic differences in prescribing.

Our study reports a 36% lower risk of an AKI-related hospitalization in those who received albumin, while the absolute difference in overall AKI rates was not statistically significant. In addition, our multivariable model did not include key clinical variables such as baseline disease severity, renal dysfunction, or comorbidities, which may influence both albumin administration and AKI outcomes. This may potentially introduce confounding by indication, particularly as patients with greater renal dysfunction might be more likely to receive albumin. Future studies must include these critical clinical variables to address this gap. While the latest AASLD practice guidance states that albumin infusion may help prevent AKI progression,^5^ there are conflicting data regarding the potential clinical benefits. For example, a recent retrospective study of patients with cirrhosis and diuretic-resistant ascites receiving outpatient albumin reported reductions in hospital admissions and improvements in biochemical markers, although only a subset underwent LVP.^37^ Meta-analyses by Bernardi et al^38^^,^^39^ in 2012 and 2014 demonstrated that albumin was linked to a 15%–19% decrease in the likelihood of ascites recurrence, renal impairment, and hospital re-admissions and a risk reduction of PPCD by 61%, hyponatremia by 42%, and mortality by 36% compared with other therapies.

However, a systematic review by Kütting et al^40^ found insufficient evidence to support a mortality benefit from albumin during LVP in HCC-free patients with cirrhosis. Similarly, Shrestha et al^41^ reported that while albumin may reduce paracentesis-induced circulatory dysfunction and hyponatremia, there was no significant effect on outcomes such as ascites recurrence, renal impairment, hepatic encephalopathy, or gastrointestinal bleeding. These findings underscore the need for further research to clarify the role of albumin beyond established indications.

Interestingly, our aGLM results report that Black and Hispanic individuals were 2.9 times and 3.5 times more likely, respectively, to have an AKI-related hospitalization within 30 days of index LVP than White individuals. This finding has clinical implications, as studies consistently indicate that patients with cirrhosis who develop AKI face higher mortality rates (34%) during hospitalization and up to 1 year post-AKI, with mortality risk increasing with AKI severity.^13^^,^^15^^,^^42^ While not statistically significant, the LVP + albumin group showed a slightly lower 30-day mortality rate, a descriptive observation that requires further investigation and should not be interpreted as clinically meaningful. Our data suggest that additional efforts are required in preventing AKI among racial and ethnic minorities with cirrhosis to mitigate complications and reduce healthcare resource utilization burden.

We also observed in our aGLM that Medicaid-insured patients were linked to a 45% reduction in the odds of an AKI-related hospitalization within 30 days of LVP. This could be attributed to the difficulties that Medicaid patients encounter, in scheduling a primary care appointment soon after a visit (ie, 30 d).^43^

This study highlights significant variations in healthcare access and clinical outcomes for cirrhosis-related complications, based on racial and ethnic backgrounds and insurance coverage. Given the rising cirrhosis rates among racial and ethnic minorities and the less educated, addressing structural racism is essential for policymakers, key stakeholders, and providers.^44^^,^^45^ Marginalized groups are underrepresented in clinical trials, yet these are the most vulnerable groups.^46^ Failing to address health literacy, social needs, and distrust will increase health disparities and worsen outcomes, underscoring the need for equitable healthcare access.

To the best of our knowledge, this is the first study to report differences in albumin administration in relation to race and ethnicity and insurance status among patients with cirrhosis and ascites undergoing LVP and the clinical implications of these practices. There are, however, limitations to conducting retrospective studies using real-world data, many of which apply to our study. First, we could not consider laboratory values such as serum albumin and MELD 3.0 due to inherent database limitations. Our multivariable model did not include these key clinical variables, which may influence both albumin administration and AKI outcomes. Despite this limitation, we have accounted for other variables indicative of clinical severity beyond laboratory values, ensuring that our findings retain significant clinical value. In addition, our dataset did not include sufficient granularity to adjust for all potential confounders, such as differences in disease severity, access to care, inpatient practice patterns, or thresholds for hospitalization, which limits the strength of causal inferences. There is a need for future research to further explore the complex interplay between payor status, treatment allocation, and clinical outcomes. Second, the purpose of LVP, whether therapeutic or diagnostic, is unclear due to ICD coding limitations. Similarly, the diagnosis of cirrhosis was based on codes rather than biopsy or noninvasive assessments. Third, data on albumin dosage and the volume of total ascitic fluid removed were not available in this dataset. However, our study included only those patients who underwent 2 LVPs within 30 days, implying that ascites was refractory and required a large volume of fluid removal. It should be noted that in other countries, such as the United Kingdom, patients undergoing 2 LVPs within 60 days are considered as those with RA and could be explored in future studies to ensure broader applicability of the findings. Furthermore, our study excluded patients who underwent LVPs for confounders, including hepatorenal syndrome, spontaneous bacterial peritonitis or other hospitalization reasons at the index encounter, as indicated by the 48-hour length of stay stipulation. Lastly, it is important to acknowledge that merging racial and ethnic groups in commercially available databases can mask important within-group differences. This practice highlights the critical need for data disaggregation to fully understand and address these differences in care among diverse racial and ethnic groups.

## CONCLUSIONS

In one of the only real-world studies evaluating albumin utilization in the context of RA, nearly 60% of patients undergoing LVPs did not receive albumin despite clinical recommendations. While patients who received albumin were found to have lower odds of AKI-related hospitalizations post-LVP, this finding reflects a correlation and not a causal relationship. It is possible that unmeasured confounders, such as MELD or Child–Pugh scores, may have influenced both albumin use and clinical outcomes. Our findings also suggest potential differences in albumin administration by race, ethnicity, and payor type. Further research is needed to clarify the drivers of these differences, including disease severity, comorbidities, access to care, and inpatient practice patterns. The study findings highlight an opportunity for broader albumin utilization in U.S. patients undergoing repeated LVPs for RA, which may potentially mitigate downstream AKI-related admissions. There is a growing interest in how health disparities and social determinants influence cirrhosis outcomes. It is crucial that staff undertake antiracism and bias training, and providers comply with guideline-based recommendations.

## Supplementary Material
