# Supplementary material for: Albumin prevents kidney injury but is underutilized in a cohort of patients undergoing large-volume paracentesis
Source: Hepatol Commun. 2025 Sep 29;9(10):e0760. doi: 10.1097/HC9.0000000000000760 (PMC12483065; doi:10.1097/HC9.0000000000000760)
Supplement: Supplementary file 1 [file hc9-9-e0760-s001.pdf]

Table S1: ICD/CPT codes for cirrhosis, and cirrhosis-related complications and procedures

Condition	Type	Code	Description
Cirrhosis	ICD-10 DX	K70.30	Alcoholic cirrhosis of liver without ascites
	ICD-10 DX	K74.0	Hepatic fibrosis
	ICD-10 DX	K74.60	Unspecified cirrhosis of liver
	ICD-10 DX	K74.69	Other cirrhosis of liver
Additional Cirrhosis Codes[1]	ICD-10 DX	I85.9	Oesophageal varices without bleeding
	ICD-10 DX	I98.2	Oesophageal varices without bleeding in diseases classified elsewhere
	ICD-10 DX	K71.7	Toxic liver disease with fibrosis and cirrhosis of liver
	CPT	81703	Complications of cirrhosis
	CPT	81803	Complications of cirrhosis
	ICD-10 DX	I85.0	Esophageal varices
	ICD-10 DX	I86.4	Gastric varices
	ICD-10 DX	I98.3	Oesophageal varices with bleeding in diseases classified elsewhere
	ICD-10 DX	R17	Unspecified jaundice
	ICD-10 DX	R18	Ascites
	CPT	Z76804	Complications of cirrhosis
Ascites/paracentesis procedures	ICD-10 DX	K70.31	Alcoholic cirrhosis of liver with ascites
	ICD-10 DX	K70.11	Alcoholic hepatitis with ascites
	ICD-10 DX	K71.51	Toxic liver disease with chronic active hepatitis with ascites
	ICD-10 DX	R18.8	Other ascites
	ICD-10 PR	0D9S30Z	Drainage of Greater Omentum with Drainage Device, Percutaneous Approach
	ICD-10 PR	0D9S3ZZ	Drainage of Greater Omentum, Percutaneous Approach
	ICD-10 PR	0D9S40Z	Drainage of Great Omentum with Drain Dev, Perc Endo Approach
	ICD-10 PR	0D9S4ZZ	Drainage of Greater Omentum, Percutaneous Endoscopic Approach
	ICD-10 PR	0D9T30Z	Drainage of Lesser Omentum with Drainage Device, Percutaneous Approach
	ICD-10 PR	0D9T3ZZ	Drainage of Lesser Omentum, Percutaneous Approach
	ICD-10 PR	0D9T40Z	Drainage of Lesser Omentum with Drainage Device, Percutaneous Endoscopic Approach
	ICD-10 PR	0D9T4ZZ	Drainage of Lesser Omentum, Percutaneous Endoscopic Approach
	ICD-10 PR	0D9V30Z	Drainage of Mesentery with Drainage Device, Percutaneous Approach
	ICD-10 PR	0D9V3ZZ	Drainage of Mesentery, Percutaneous Approach
	ICD-10 PR	0D9V40Z	Drainage of Mesentery with Drainage Device, Percutaneous Endoscopic Approach
	ICD-10 PR	0D9V4ZZ	Drainage of Mesentery, Percutaneous Endoscopic Approach
	ICD-10 PR	0D9W30Z	Drainage of Peritoneum with Drainage Device, Percutaneous Approach
	ICD-10 PR	0D9W3ZZ	Drainage of Peritoneum, Percutaneous Approach
	ICD-10 PR	0D9W40Z	Drainage of Peritoneum with Drainage Device, Percutaneous Endoscopic Approach
	ICD-10 PR	0D9W4ZZ	Drainage of Peritoneum, Percutaneous Endoscopic Approach
	ICD-10 PR	0W9F30Z	Drainage of Abdominal Wall with Drainage Device, Percutaneous Approach
	ICD-10 PR	0W9F3ZZ	Drainage of Abdominal Wall, Percutaneous Approach
	ICD-10 PR	0W9F40Z	Drainage of Abdominal Wall with Drainage Device, Percutaneous Endoscopic Approach
	ICD-10 PR	0W9F4ZZ	Drainage of Abdominal Wall, Percutaneous Endoscopic Approach
	ICD-10 PR	0W9G30Z	Drainage of Peritoneal Cavity with Drainage Device, Percutaneous Approach
	ICD-10 PR	0W9G3ZZ	Drainage of Peritoneal Cavity, Percutaneous Approach
	ICD-10 PR	0W9G40Z	Drainage of Peritoneal Cavity with Drainage Device, Percutaneous Endoscopic Approach
	ICD-10 PR	0W9G4ZZ	Drainage of Peritoneal Cavity, Percutaneous Endoscopic Approach
	ICD-10 PR	0W9J30Z	Drainage of Pelvic Cavity with Drainage Device, Percutaneous Approach
	ICD-10 PR	0W9J3ZZ	Drainage of Pelvic Cavity, Percutaneous Approach
	CPT	49080	Peritoneocentesis, abdominal paracentesis or peritoneal lavage (diagnostic or therapeutic); initial
	CPT	49081	Peritoneocentesis, abdominal paracentesis or peritoneal lavage (diagnostic or therapeutic); subsequent
AKI	ICD-10 DX	N17.0	Acute kidney failure with tubular necrosis
	ICD-10 DX	N17.1	Acute kidney failure with acute cortical necrosis
	ICD-10 DX	N17.2	Acute kidney failure with medullary necrosis
	ICD-10 DX	N17.8	Other acute kidney failure
	ICD-10 DX	N17.9	Acute kidney failure, unspecified
	ICD-10 DX	N99.0	Postprocedural (acute) (chronic) kidney failure
AKI (RRT)	ICD-10 DX	T82.41XA	Breakdown (mechanical) of vascular dialysis catheter, initial encounter
	ICD-10 DX	T82.42XA	Displacement of vascular dialysis catheter, initial encounter
	ICD-10 DX	T82.49XA	Other complication of vascular dialysis catheter, initial encounter
	ICD-10 DX	Z49.01	Encounter for fitting and adjustment of extracorporeal dialysis catheter
	ICD-10 PR	05HY33Z	Insertion of Infusion Device into Upper Vein, Percutaneous Approach
	ICD-10 PR	06HY33Z	Insertion of Infusion Device into Lower Vein, Percutaneous Approach
	ICD-10 PR	5A1D00Z	Performance of Urinary Filtration, Single

	ICD-10 PR	5A1D60Z	Performance of Urinary Filtration, Multiple
	ICD-10 PR	5A1D70Z	Performance of Urinary Filtration, Intermittent, Less than 6 Hours Per Day
	ICD-10 PR	5A1D80Z	Performance of Urinary Filtration, Prolonged Intermittent, 6-18 hours Per Day
	ICD-10 PR	5A1D90Z	Performance of Urinary Filtration, Continuous, Greater than 18 hours Per Day
	ICD-10 PR	3E1M39Z	Irrigation of Peritoneal Cavity using Dialysate, Percutaneous Approach
Additional AKI codes identified from[2-4]	ICD-10 DX	N14.1	Nephropathy induced by other drugs, medicaments and biological substances
	ICD-10 DX	N14.2	Nephropathy induced by unspecified drug, medicament or biological substance
	ICD-10 DX	N19	Unspecified kidney failure
	ICD-10 DX	R34	Anuria and oliguria
	ICD-10 DX	R94.4	Abnormal results of kidney function studies
	CPT	G0491	Dialysis procedure at a medicare certified ESRD facility for acute kidney injury without ESRD
Liver transplant	ICD-10 DX	T86.40	Unspecified complication of liver transplant
	ICD-10 DX	T86.41	Liver transplant rejection
	ICD-10 DX	T86.42	Liver transplant failure
	ICD-10 PR	0FY00Z0	Transplantation of Liver, Allogeneic, Open Approach
	ICD-10 PR	0FY00Z1	Transplantation of Liver, Syngeneic, Open Approach
	ICD-10 PR	0FY00Z2	Transplantation of Liver, Zooplastic, Open Approach
	CPT	47135	Liver allotransplantation; orthotopic, partial or whole, from cadaver or living donor, any age
	CPT	47136	Liver allotransplantation; heterotopic, partial or whole, from cadaver or living donor, any age
Hepatic encephalopathy	ICD-10 DX	K72.09	Hepatic failure, unspecified
	ICD-10 DX	K72.91	Hepatic failure, unspecified with coma
Hepatitis C	ICD-10 DX	B18.2	Chronic viral hepatitis C
	ICD-10 DX	B17.10	Acute hepatitis C without hepatic coma
	ICD-10 DX	B19.21	Unspecified viral hepatitis C with hepatic coma
	ICD-10 DX	B17.11	Acute hepatitis C with hepatic coma
Hepatitis B	ICD-10 DX	B19.10	Unspecified viral hepatitis B without hepatic coma
	ICD-10 DX	B18.1	Chronic viral hepatitis B without delta-agent
	ICD-10 DX	B16.9	Acute hepatitis B without delta-agent and without hepatic coma
	ICD-10 DX	B19.11	Unspecified viral hepatitis B with hepatic coma
	ICD-10 DX	B16	Acute hepatitis B
	ICD-10 DX	B17.0	Acute delta-(super) infection of hepatitis B carrier
	ICD-10 DX	B18.0	Chronic viral hepatitis B with delta-agent
MASLD	ICD-10 DX	K76.9	Liver disease, unspecified
	ICD-10 DX	K76.0	Fatty (change of) liver, not elsewhere classified
	ICD-10 DX	K76.89	Other specified diseases of liver
MASH	ICD-10 DX	K75.81	Nonalcoholic steatohepatitis (NASH)

Abbreviations: AKI = Acute kidney injury, CPT = Current procedural terminology, ESRD = End-stage renal disease, ICD-10 = International Classification of Disease Tenth Revision, LVP = Large volume paracentesis, MASLD = Metabolic dysfunction-associated steatotic liver disease, MASH = Metabolic dysfunction-associated steatohepatitis, RRT = Renal replacement therapy

References

1. Lapointe-Shaw L, Georgie F, Carlone D, et al. Identifying cirrhosis, decompensated cirrhosis and hepatocellular carcinoma in health administrative data: A validation study. *PLoS One*. Aug 2018;13(8):e0201120. doi: 10.1371/journal.pone.0201120.
2. Sun H, Depraetere K, Meesseman L, et al. A scalable approach for developing clinical risk prediction applications in different hospitals. *J Biomed Inform*. Jun 2021;118:103783. doi: 10.1016/j.jbi.2021.103783.
3. Mansfield KE, Nitsch D, Smeeth L, Bhaskaran K, Tomlinson LA. Prescription of renin-angiotensin system blockers and risk of acute kidney injury: a population-based cohort study. *BMJ Open*. Dec 2016;6(12):e012690. doi: 10.1136/bmjopen-2016-012690.
4. American Nephrology Nurses Association. Acute Kidney Injury Fact Sheet: Financial/Payment/Billing Issues. Published 2022. Accessed July 31, 2025. https://www.annanurse.org/wp-content/uploads/2025/04/akiFinancialFactSheet.pdf
